# Finger-to-Beat Coordination Skill of Non-dancers, Street Dancers, and the World Champion of a Street-Dance Competition

**DOI:** 10.3389/fpsyg.2016.00542

**Published:** 2016-04-20

**Authors:** Akito Miura, Shinya Fujii, Masahiro Okano, Kazutoshi Kudo, Kimitaka Nakazawa

**Affiliations:** ^1^Faculty of Sport Sciences, Waseda UniversityTokorozawa, Japan; ^2^Graduate School of Education, The University of TokyoTokyo, Japan; ^3^Department of Life Sciences, Graduate School of Arts and Sciences, The University of TokyoTokyo, Japan

**Keywords:** dynamical systems approach, sensorimotor synchronization, sensorimotor learning, rhythm, dance

## Abstract

The coordination of body movements to a musical beat is a common feature of many dance styles. However, the auditory–motor coordination skills of dancers remain largely uninvestigated. The purpose of this study was to examine the auditory–motor coordination skills of non-dancers, street dancers, and the winner of a celebrated international street dance competition, while coordinating their rhythmic finger movements to a beat. The beat rate of a metronome increased from 1.0 to 3.7 Hz. The participants were asked to either flex or extend their index fingers on the beat in each condition. Under the extend-on-the-beat condition, both the dancers and non-dancers showed a spontaneous transition from the extend-on-the-beat to the flex-on-the-beat or to a phase wandering pattern. However, the critical frequency at which the transition occurred was significantly higher in the dancers (3.3 Hz) than in the non-dancers (2.6 Hz). Under the flex-on-the-beat condition, the dancers were able to maintain their coordination pattern more stably at high beat rates compared to the non-dancers. Furthermore, the world champion matched the timing of movement peak velocity to the beat across the different beat rates. This may give a sense of unity between the movement and the beat for the audience because the peak velocity of the rhythmic movement works as a temporal cue for the audiovisual synchrony perception. These results suggest that the skills of accomplished dancers lie in their small finger movements and that the sensorimotor learning of street dance is characterized by a stabilization of the coordination patterns, including the inhibition of an unintentional transition to other coordination patterns.

## Introduction

The coordination of body movements to music is a common feature of many dance styles, including folk, ballet, modern, social, jazz, and street dance. Accomplished dancers coordinate their body movements in a highly organized fashion. Within the last decade, researchers in performance science have focused on a broad range of issues impacting dance performance, including medical problems (Hincapié et al., 2008), the fitness levels of dancers, such as cardiorespiratory property (Wyon, 2005), muscle strength (Koutedakis et al., 2005), body composition (Wilmerding et al., 2005), and flexibility (Deighan, 2005). However, dancers’ auditory–motor coordination^1^ (AMC) skills (i.e., the sensorimotor skills to coordinate their movements to sounds, such as a rhythmic metronome and musical beat) remain largely unknown and under-investigated. The performance science of dancers’ AMC skills is fundamental for a better understanding of accomplished dance performances and for the development of effective dance training methods. AMC skills are an explicit sign of adaptive plasticity of the central nervous system (for a review, Repp and Su, 2013). Comparing the AMC between dancers and non-dancers affords a means of investigating this adaptive plasticity, especially in dancers with long-term practice over several years (for a review on dancers’ brain, Karpati et al., 2015). In this paper, we focused on street dance^2^ because research on street dancers (Miura et al., 2011, 2013a) revealed an enhanced AMC skill, suggesting that they are a suitable population to investigate the plasticity of AMC. The purpose of this study is to further investigate the AMC skill in street dancers. Hereafter, we refer to street dancers as dancers.

To elucidate a dancer’s AMC skills, the dynamical systems approach (DSA) is thought to be useful (for a review, Miura et al., 2015). The DSA was originally developed to describe the synchronization^1^ patterns in nature and the changes over time (e.g., the synchronization of flashing light patterns in fireflies; Buck, 1988; Strogatz, 2003). In Kelso (1984), the DSA was applied to human movement coordination. Specifically, the DSA was used to describe the coordination patterns of rhythmic bimanual finger movements (Kelso, 1984). In an experiment reported by Kelso (1984), an individual was asked to rhythmically abduct/adduct their index fingers under two different initial conditions: one in which the homologous muscles were activated in synchrony (i.e., an in-phase pattern) and the other in which the homologous muscles were alternatively activated (i.e., the anti-phase pattern). When the individual started the anti-phase pattern, a spontaneous phase transition from the anti-phase to the in-phase pattern occurred at a critical frequency as the movement frequency increased (Kelso, 1984). Thus, the human movement system has an intrinsic tendency towards a certain pattern (e.g., the in-phase pattern in the previously described case) at high movement frequencies. This tendency is termed the intrinsic coordination tendency in the DSA. Haken et al. (1985) formulated a mathematical model to describe this intrinsic coordination tendency and phase transition phenomenon. Their model is called the Haken–Kelso–Bunz (HKB) model, named after their initials. The HKB model has been applied to various types of human movement coordinations, such as rhythmic inter-limb coordination (Schöner et al., 1986; Kelso and Jeka, 1992; Jeka and Kelso, 1995; Fujii et al., 2010), inter-personal coordination (Schmidt et al., 1990; Schmidt and Turvey, 1994; Alderisio et al., 2015), and action-perception coordination (Kelso et al., 1990).

Recently, the phase transition phenomenon was found in the AMC during street dance (Miura et al., 2011, 2013a). During street dance, there are two coordination patterns when an individual synchronizes their knee movements to a musical beat in a stance: down-on-the-beat and up-on-the-beat. The down-on-the-beat is the pattern in which an individual flexes their knees on the beat, while the up-on-the-beat is the pattern in which the dancers extend their knees on the beat. This task is familiar to street dancers because it is a basic technique. Miura et al. (2013a) found that both street dancers and non-dancers showed an unintentional phase transition from the up-on-the-beat to the down-on-the-beat pattern when they were performing the up-on-the-beat at high beat rates. However, the critical frequency where the phase transition occurred was significantly higher in the street dancers (2.8 Hz) than in the non-dancers (2.1 Hz). In addition, when the dancers were asked to resist the unintentional phase transition to the down-on-the-beat, a skilled street dancer did not show an unintentional phase transition and maintained the up-on-the-beat pattern at 3.0 Hz (Miura et al., 2011). These findings suggest that the street dancers had modified the intrinsic coordination tendency through dance practice, such that they were able to maintain the up-on-the-beat pattern at high beat rates.

Although Miura et al. (2011, 2013a) have investigated dancer’s AMC skills, there are at least two issues that need to be clarified. First, it remains unknown whether the dancer’s AMC skills are task-dependent. Miura et al. (2011, 2013a) used the task to coordinate the knee movements to the musical beat. The question then arises as to whether these results are consistent if dancers only move the distal part of their body, such as a finger, without moving other body parts. The German-born architect, Ludwig Mies van der Rohe, said, “God is in the details”, which means that the details are important in design and art. If this pertains to dance performances, the skills of accomplished dancers lie not only in their big knee movements (Miura et al., 2011, 2013a,b, 2014), but also in their small finger movements. Interestingly, a previous study by Kelso and Zanone (2002) demonstrated a transfer of the learning effect among the motor effectors. After learning a novel inter-limb coordination pattern with a set of motor effectors (e.g., legs), the learning effect was transferred to another set of untrained motor effectors (e.g., arms). These results suggest that coordination learning is effector-independent and that a high-level neural representation might govern coordination dynamics (Kelso and Zanone, 2002). If so, the learning effect that is gained from dance practice to coordinate the knee movements may be transferred to the finger coordination to a beat, and vice versa. Thus, we assumed that the intrinsic coordination tendency and the modification of that by street dance training during the knee-to-beat coordination may be similar to those that are present during the finger-to-beat coordination. However, to our knowledge, a dancer’s ability to coordinate their finger movements to a beat has not been clarified.

Second, there has been no investigation of the AMC skills of a winner of a celebrated international street dance competition. The performance of a world champion in sports and music fascinates not only the general population but also researchers, since it provides an opportunity to elucidate finely tuned neuromuscular adaptations in humans (Fujii et al., 2009; Fujii and Moritani, 2012a,b). For example, the previous studies on the world fastest drummer showed that the performance was significantly different compared to other drummers (Fujii et al., 2009; Fujii and Moritani, 2012a,b). This indicates that individual differences can be large, even within a group of skilled performers. Thus, it is important to analyze the individual level in performance science. Taken together, we need to investigate how the finger-to-beat coordination skills of dancers, especially the skill of a world champion, are different from that of non-dancers.

Considering this context, we examined the coordination of the finger movements to a beat in non-dancers, street dancers, and a world champion. Consistent with the findings of Miura et al. (2013a), we hypothesized that the dancers would be able to maintain the less stable coordination pattern (i.e., the extend-on-the-beat pattern) at high beat rates compared to that of non-dancers while coordinating their finger movements to a metronome beat. To test this, we used the phase transition paradigm of the finger-to-beat coordination task (Kelso et al., 1990; Carson, 1996) with the same increasing beat rate as the Miura et al.’s (2013a) study. We compared the dancers and non-dancers to test our hypothesis. We also discussed the finger-to-beat coordination skills of a world champion.

## Materials and Methods

### Participants

Six skilled street dancers and six controls participated in this experiment. The street dancers (men; age: 27.8 ± 5.8 years [mean ± standard deviation (SD)]) had 8427 ± 3039 h of accumulated dance practice time (11.2 ± 6.0 years of dancing experience). One of the dancers won a celebrated international street dance competition. Hereafter, we refer to this individual as the world champion. The other five participants were not street dancing at an international level, but at a national level. The novice non-dancer controls (men; age: 26.0 ± 2.4 years of age) had no experience in any kind of dance. Hereafter, we refer to the dancer group as dancers and the control group as non-dancers. Five non-dancers were athletes at their college/university, and one was an athlete in his high school. Most non-dancers had been enjoying physical activities at a recreational level on a regular basis after they stopped playing their sports as active athletes. Thus, the fitness level appeared not to be different between the groups. We counterbalanced the number of participants who had experience of practicing musical instruments: four dancers and four non-dancers had experience of practicing musical instruments. All participants were consistently right-handed and had no history of a handedness correction from left to right-handedness. The handedness was accessed by the Edinburgh Inventory (Oldfield, 1971). The mean handedness score was 78.1 ± 14.7 and 92.9 ± 8.4 in dancers and non-dancers, respectively. The handedness score of the groups tended to be different (ranked Welch’s test, *p* = 0.0502). Informed consent was obtained from all participants for their participation in the study. This study was approved by the Ethics Committee of the Graduate School of Arts and Sciences, the University of Tokyo.

### Task and Design

**FIGURE 1:**
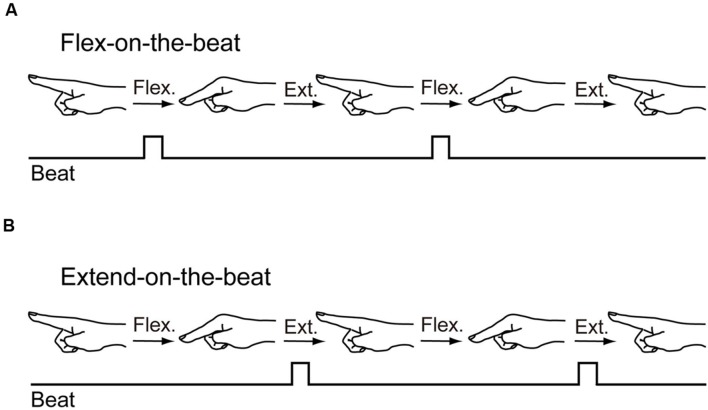
**Two patterns in the finger-to-beat coordination task: flex-on-the-beat pattern (A) and extend-on-the-beat pattern (B)**.

The participants flexed their right elbow joint to 90° in a standing posture. The right forearm rested on a horizontal platform. The right wrist joint was fixed to the platform with a tape to prevent any wrist joint movement. The participants extended their right index finger while the remaining fingers were curled into a fist with their thumb inside. There were two patterns in the finger-to-beat coordination task: flex-on-the-beat and extend-on-the-beat. During the flex-on-the-beat pattern, the individual flexed their finger on the beat. During the extend-on-the-beat pattern, the individual extended their finger on the beat (**Figure 1**). The range of motion was not specified. The beat rate increased from 1.0 to 3.7 Hz, with a step of 0.33 Hz (i.e., 1.0, 1.3, 1.7, 2.0, 2.3, 2.7, 3.0, 3.3, and 3.7 Hz). There were nine beat rates in total; the beat rate is shown to the first decimal place by rounding the second decimal place. Each of the beat rate plateaus consisted of 16 beats (144 beats in total). After 144 beats, the metronome automatically stopped and the participants stopped the movement. A trial took 75 s and the data across the 144 beats were recorded. This beat rate manipulation was same as the Miura et al.’s (2013a) study.

The task consisted of two experimental conditions. The participants initiated the flex-on-the-beat pattern in one condition and initiated the extend-on-the-beat pattern in the other condition. They were instructed as follows: “Please look straight and don’t look at your finger. Please flex (extend) your index finger at the metacarpophalangeal joint to a metronome beat. The beat rates will increase gradually. Try to keep a 1:1 relationship between the metronome beat and the rhythmic movement. Don’t intervene in the pattern change, that is, there is no problem if your coordination pattern changes from flex(extend)-on-the-beat to extend(flex)-on-the-beat. The range of motion is up to you, move your finger within a comfortable range. Don’t count the number of metronome beats.” Each participant repeated the two experimental conditions three times in a random order (6 trials in total). In order to avoid fatigue, the participants were allowed to have a rest between the trials. The rest duration was at least a few minutes or more, to the extent that they did not experience any fatigue or discomfort.

### Apparatus and Data Collection

The angular displacement was measured by an electrogoniometer (Biopac Systems, Tokyo, Japan) that was attached over the metacarpophalangeal joint of the right index finger. The electrogoniometer signal was sampled at 1000 Hz with a MP100 recording system (Biopac Systems). The data were recorded on a personal computer with wave recording/analyzing software (AcqKnowledge 3.7.3 for Windows; Biopac Systems). The metronome beat was programmed with a programmable metronome, DR-880 Dr. Rhythm (Roland Corporation, Shizuoka, Japan).

### Data Analysis

The angular displacement data were smoothed using a forward and back 2nd order Butterworth low-pass filter with a cut-off frequency of 10 Hz. Here, the data value became smaller as the finger flexed, and became larger when the finger extended. We calculated the angular velocity by differentiating the angular displacement.

**FIGURE 2:**
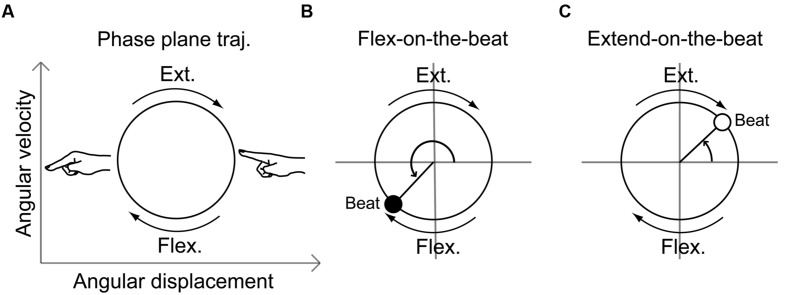
**Schematic illustration of the movement trajectory on a phase plane (A) and the phase angle at a beat onset time during the flex-on-the-beat pattern (B) and the extend-on-the-beat pattern (C).** The phase plane is composed of the angular displacement and the angular velocity.

The angular displacement and angular velocity data were segmented for each beat rate plateau. The segmented data were converted to *Z*-values and the linear trend was removed. The angular displacement data were then plotted against the angular velocity data (**Figure 2A**). This plot is referred to as the phase plane and the plotted line is termed the movement trajectory. Then, the beat onset time was superposed on the movement trajectory (Miura et al., 2011, 2013a). To assess the coordination relationship between the finger movement and the beat, we calculated the phase angle of the movement trajectory at the beat onset time. If the beat onset time was located between the phase angle of 0–180° (**Figure 2C**), the beat took place during the finger extension phase. If it was located between the phase angle of 180°–360° (**Figure 2B**), the beat took place during the finger flexion phase. We calculated the mean and SD of the movement phase angles at the beat time using circular statistics (Batschelet, 1981).

Consistent with the Miura et al.’s (2013a) study, we determined the critical frequency at which the spontaneous transition from the extend-on-the-beat to the flex-on-the-beat or to the phase wandering occurred. The phase wandering refers to a pattern in which the movement frequency did not match with a beat rate, resulting in a lack of synchronization (e.g., a finger could not move faster than a metronome at a high tempo). To determine the critical frequency, we first calculated the mean phase angle across all beat rates and across three trials in the flex-on-the-beat condition for each participant. The mean phase angles were further averaged across all the participants. We defined the grand average ± 90° range (223 ± 90°) as the flex-on-the-beat range. Note that this grand average was calculated by averaging the data from all participants because we found no significant differences between the groups in the mean phase angle (see Section “Results” below). We defined the critical frequency as the beat rate at which the three consecutive phase angles got in the flex-on-the-beat range under the extend-on-the-beat condition. Some of the phase wandering patterns did not meet this criterion because the wandering occurred within the three consecutive phase angles. In such cases, we defined the critical frequency as the beat rate at which the difference between the maximum and minimum phase angles within the beat rate was greater than 540°.

### Statistical Analysis

We performed a two-way analysis of variance (ANOVA) with a between-subject factor of group (dancer vs. non-dancer) and a within-subject factor of beat rate (1.0, 1.3, 1.7, 2.0, 2.3, 2.7, 3.0, 3.3, and 3.7 Hz) on (i) the mean and (ii) the SD of the phase angle of the movement at the beat time. When the Mauchly’s test of sphericity showed a heterogeneity of covariance, the more conservative Greenhouse–Geisser test was performed in the ANOVA. The unpaired Welch’s test was used for the critical frequency to compare the dancers and non-dancers. The level of statistical significance was set at *p* < 0.05.

## Results

### Mean Phase Angle

**FIGURE 3:**
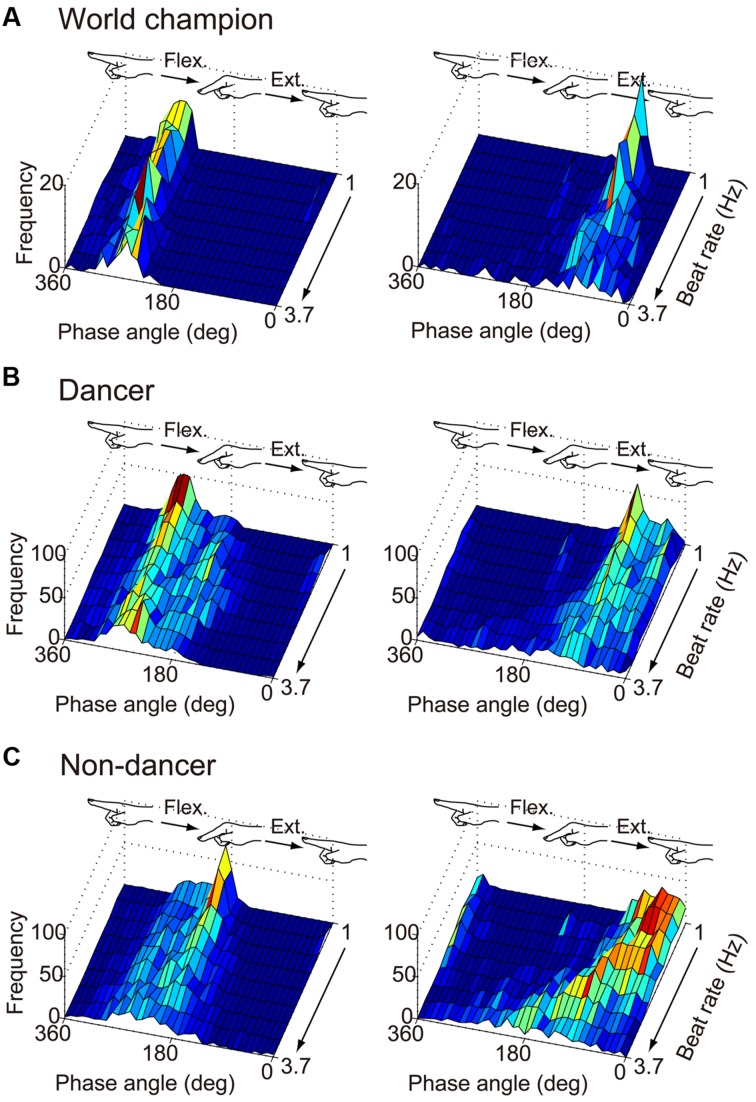
**Frequency of the phase angle of the movement trajectory at the beat onset time of the world champion (A), dancers (B), and non-dancers (C).** The left column is under the flex-on-the-beat condition, while the right is under the extend-on-the-beat condition. The data of dancers include the world champion.

**FIGURE 4:**
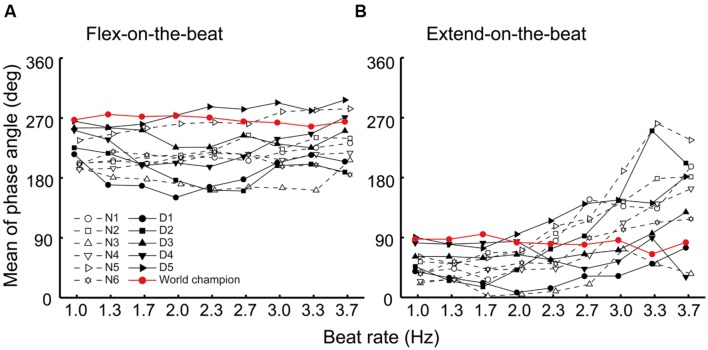
**Mean phase angle of the movement trajectory at the beat time under the flex-on-the-beat (A) and the extend-on-the-beat (B) conditions.** The data were plotted for each participant. The dashed line denotes the non-dancers, the solid line denotes the dancers, and the red line denotes the world champion.

**FIGURE 5:**
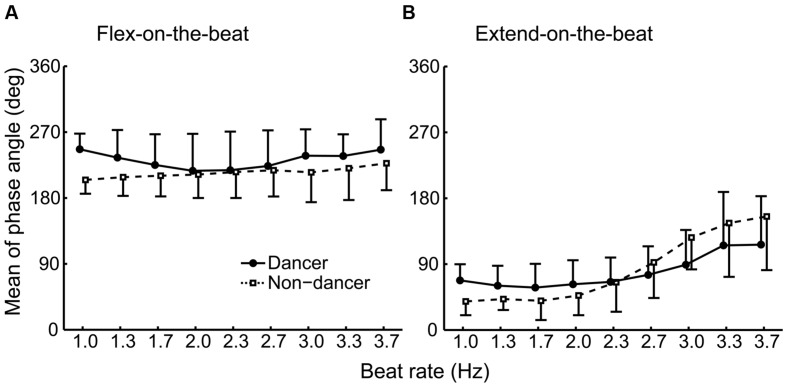
**Group average of the mean phase angle under the flex-on-the-beat (A) and the extend-on-the-beat (B) conditions.** The dashed line denotes the non-dancers, and the solid line denotes the dancers. The vertical bars represent the between-subject standard deviations (SDs). The data of dancers include the world champion.

**Figure 3** shows the distribution of the phase angle at the beat time for the world champion, dancers and non-dancers. **Figure 4** shows the individual plot of the mean phase angle and **Figure 5** shows the group average. For the flex-on-the-beat condition, the two-way ANOVA revealed no significant interaction between the factors of group and beat rate: *F*_(3.066,30.657)_ = 2.005, *p* = 0.133. No significant main effect of group nor beat rate was found based on ANOVA: *F*_(1,10)_ = 0.791, *p* = 0.395; *F*_(3.066,30.657)_ = 2.518, *p* = 0.075, respectively. For the extend-on-the-beat condition, the interaction was also not significant: *F*_(1.510,15.097)_ = 2.107, *p* = 0.163. There was no significant main effect of group, *F*_(1,10)_ = 0.065, *p* = 0.804. There was significant main effect of beat rate, *F*_(1.510,15.097)_ = 14.701, *p* = 0.001.

### Variability of the Phase Angle

**FIGURE 6:**
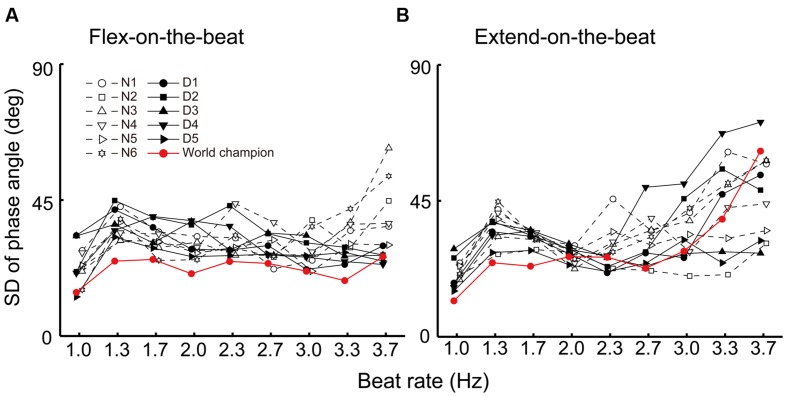
**Standard deviation of the phase angle under the flex-on-the-beat (A) and the extend-on-the-beat (B) conditions.** The data are plotted for each participant. The dashed line denotes the non-dancers, the solid line denotes the dancers, and the red line denotes the world champion.

**FIGURE 7:**
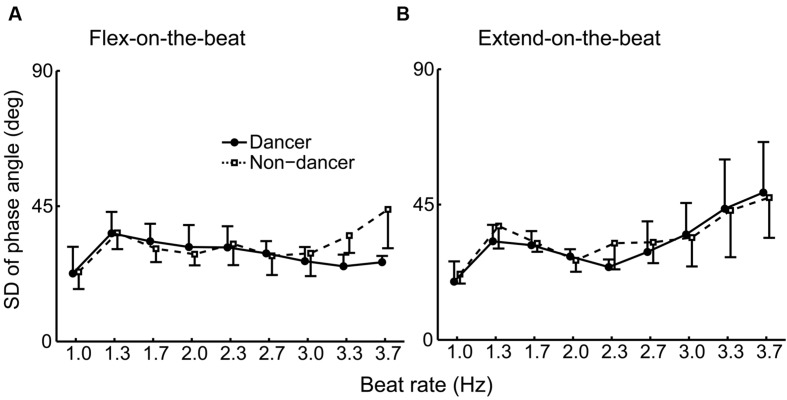
**Group average of the SD of the phase angle under the flex-on-the-beat (A) and the extend-on-the-beat (B) conditions.** The dashed line denotes the non-dancers, and the solid line denotes the dancers. The vertical bars represent the between-subject SDs. The data of dancers include the world champion.

**Figure 6** shows the SD of the phase angle at the beat time for each participant and **Figure 7** shows the group average. For the flex-on-the-beat condition, the ANOVA on the SD of the phase angle found a significant interaction between the factors of group and beat rate: *F*_(8,80)_ = 4.592, *p* < 0.001. Thus, the unpaired Welch’s tests were performed to compare the groups at each beat rate. The test showed that the SD of the phase angle of the non-dancers was significantly larger than that of the dancers at the high beat rates (3.3 and 3.7 Hz; *p* = 0.002 and 0.015, respectively). For the extend-on-the-beat condition, the ANOVA showed no significant interaction: *F*_(1.949,19.491)_ = 0.608, *p* = 0.551. The main effect of group was not significant: *F*_(1,10)_ = 0.243, *p* = 0.632. The main effect of the beat rate was significant, showing that the variability of the phase angle became larger as the beat rate increased: *F*_(1.949,19.491)_ = 15.523, *p* < 0.001.

### Critical Frequency

**Table 1** summarizes the critical frequencies of all participants. Three participants (N3, D1, and D3) did not show a transition from the extend-on-the-beat pattern to the other patterns in one of the three trials. The mean critical frequency was 3.3 Hz in the dancers and 2.6 Hz in the non-dancers. The unpaired Welch’s test revealed a significant difference between the groups (*p* = 0.010). Thus, the critical frequency in the dancers was significantly higher than that in the non-dancers.

**Table 1:** Critical frequency where the transition from the extend-on-the-beat pattern to the flex-on-the-beat pattern or to the phase wandering pattern occurred.

	Set 1	Set 2	Set 3	Average
**Non-dancer**				
N1	1.3	2.7	3.0	2.3
N2	2.7	3.0	2.7	2.8
N3	3.7	No transition	2.3^∗^	3.0
N4	3.0	3.3	1.7	2.7
N5	3.0	2.3	3.0	2.8
N6	2.7	1.3	3.0^∗^	2.3

Average ± SD				2.6 ± 0.27

**Dancer**				
D1	3.7	No transition	3.7	3.7
D2	3.0	3.0	3.0	3.0
D3	No transition	3.7	3.3	3.5
D4	3.3^∗^	3.3^∗^	3.0^∗^	3.2
D5	2.7	2.7	2.7	2.7
World champion	3.7^∗^	3.3^∗^	3.7	3.6

Average ± SD				3.3 ± 0.38

*Asterisk indicates transition to phase wandering pattern.*

## Discussion

The purpose of this study was to examine the abilities of non-dancers, street dancers, and a world champion to coordinate their finger movements to a beat. We hypothesized that the dancers would maintain the less stable coordination pattern (i.e., the extend-on-the-beat pattern) at high beat rates compared to that of the non-dancers. Our results support this hypothesis. The dancers had a significantly higher critical frequency than the non-dancers had during the extend-on-the-beat condition. In addition, we found that the dancers showed less variability of the phase angle than the non-dancers did during the flex-on-the-beat condition at the high beat rates (i.e., 3.3 and 3.7 Hz). Thus, the dancers were able to maintain the prescribed coordination patterns more stably at high beat rates during the finger-to-beat coordination task. The prevention of the unintentional transition at high movement frequencies enables intentional switches from a coordination pattern to other patterns at the selected frequencies (Scholz et al., 1987; Scholz and Kelso, 1990). Thus, the stabilization of the coordination patterns at high movement frequencies is thought to be an indication of a diverse expression in street dance.

There are two possible interpretations of the present results. The first is that the dancers acquired the AMC skills irrespective of the motor effectors. In the previous study by Miura et al. (2011, 2013a), the dancers were able to maintain prescribed coordination patterns at high beat rates during the knee-to-beat coordination task. This is consistent with the present study. Kelso and Zanone (2002) suggested that what is learned during coordination learning is effector-independent, and that a high level neural representation enables the learning transfer from a set of trained effectors (e.g., legs) to another set of untrained effectors (e.g., arms). Considering this, we suggest that the practice of coordination between the movement of a body part (e.g., finger or knee) and the beat in street dance may modify the intrinsic coordination tendency irrespective of the motor effectors, that is, there may be common neural representation shared by different motor effectors during the AMC. Nevertheless, we cannot rule out the second interpretation that the present findings are simply due to the practice of the AMC of fingers that is needed in street dance. Future longitudinal studies are needed to clarify whether the practice of the knee/finger-to-beat coordination in dance mutually affect each other.

### Difference between Knee-to-Beat vs. Finger-to-Beat Coordination

While the results of this study were consistent with those of a previous study by Miura et al. (2013a) in terms of the advanced coordination skills of dancers, the critical frequency in this study was higher overall compared to that of the Miura et al.’s (2013a) study. In this study, the average critical frequency was 3.3 Hz in the dancers and 2.6 Hz in the non-dancers for the finger-to-beat coordination. In the study by Miura et al., the average critical frequency was 2.8 Hz in the dancers and 2.1 Hz in the non-dancers for the knee-to-beat coordination. Thus, the critical frequency in the finger-to-beat coordination was higher overall than that in the knee-to-beat coordination. This discrepancy may be attributed to the difference in the natural frequency between a finger and the whole body. The natural frequency of a finger is higher than that of the whole body because of the differences in their mechanical properties. Therefore, the coupling between a metronome and finger oscillation is thought to be less “de-tuned” compared to that between a metronome and the whole body. In fact, previous studies have shown that the de-tuning of coupled oscillators can lead to a loss of stability in a system and a phase wandering pattern (Kelso et al., 1990; Fujii et al., 2010). Taken together, de-tuning may explain why the critical frequency in this study was higher than that in the study by Miura et al. (2013a).

### Performance of the World Champion

Although the critical frequency of the world champion was the second largest among the participants, he still showed a transition from the extend-on-the-beat pattern to the flex-on-the-beat or phase wandering patterns (**Table 1**). Thus, the critical frequency measure in this study could not clearly differentiate the world champion from the other national level street dancers. In this study, the participants were instructed *not* to resist to the phase transition. This instruction could be the reason why the critical frequency measure did not differentiate between the world champion and the others. If the participants were instructed to intentionally resist the phase transition, the results may have been different. The world champion may not have exhibited the phase transition if he had resisted it intentionally. In contrast, the national level dancers may have shown the phase transition, even if they had resisted. Future studies are needed to evaluate this possibility.

In this study, the world champion showed a relatively small SD of the phase of angle across all beat rates under the flex-on-the-beat condition (**Figure 6A**). Under the extend-on-the-beat condition, the world champion showed the least amount of SD of the phase angle at the beat rates of 1.0, 1.3, and 1.7 Hz (**Figure 6B**). Although the stability of the phase angle was not least at the higher beat rates (**Figure 6B**), the world champion maintained the prescribed coordination pattern at the mean phase angle of approximately 90° across the different beat rates under the extend-on-the-beat condition (**Figure 3A** right column and **Figure 4B**). In contrast, as the beat rate increased, most of the other participants increased their variability of the phase angle (**Figure 6B**) and also changed their mean phase angle towards the flex-on-the-beat range (**Figure 4B**).

It is noteworthy that the mean phase angle of the world champion clustered around 270° under the flex-on-the-beat condition (**Figure 4A**) and around 90° under the extend-on-the-beat conditions across all beat rates (**Figure 4B**). The mean phase angles of 90 and 270° are the time points of the peak positive and negative velocities, respectively. That is, the world champion matched the peak velocity point to the beat. Su (2014) reported that the peak velocity of the rhythmically moving visual stimulus works as a temporal cue for audiovisual synchrony perception, suggesting that people feel a “visual beat” when the velocity of the rhythmic movement reached its peak value. If so, from the point of view of a dance performance, the audience may enjoy the synchronization between the “visual beat” and the musical beats when the peak velocity time was matched with the auditory beat. The mean phase angle of the world champion may represent an established sense of unity between the movements and musical beats. This would be one of the abilities that results in a compelling performance by the world champion.

Moreover, the mean phase angle of the world champion is noteworthy in terms of consistency across all beat rates. In comparison to this, the mean phase angles of other dancers and the non-dancers were not consistent across all beat rates under flex-on-the-beat condition, and across the beat rates before phase transition under extend-on-the-beat condition (**Figure 4**). In this experiment, the beat rate increased in a step-wise manner. The participants had to change the frequency of movement immediately after the metronome frequency changed. Thus, the jump in the metronome frequency worked as a perturbation because it disrupted a regular relationship between the movement and the beat (i.e., the phase angle). This may be a reason for the inconsistency of the phase angle across the beat rates in participants other than the world champion. Notably, the world champion maintained the same phase angle (i.e., 90 or 270°) before and after the jump in the metronome frequency. This suggests that the auditory–motor system of the world champion is robust to perturbations. One of the street dance techniques is to change a movement tempo quickly according to a change of musical tempo or metrical structure. The mean phase angle of the world champion might reflect the capability of such street dance technique.

Previous studies have reported that expert performance can be accomplished by deliberate practice for at least 10 years (Ericsson et al., 1993). The world champion in this study reported that his accumulated dance practice consisted of 13,832 h (19 years of experience). The number of practice hours of the world champion was the highest among the dancers group. If the amount of dance practice is related to the performance of finger-to-beat coordination task, there should be a close relationship between the number of hours of dance practice and the coordination measures within the dancers. We calculated the root mean square error (RMSE) of the mean phase angle from the peak velocity point (i.e., 90° under extend-on-the-beat condition and 270° under flex-on-the-beat condition) because the mean phase angle of the world champion clustered around the peak velocity points. Pearson’s correlations were used to explore the relationship between the dance practice hours and (i) the RMSE of the mean phase angle, (ii) the SD of the phase angle at 3.3 and 3.7 Hz under flex-on-the-beat condition (these frequencies were chosen because there were significant differences between the groups at these frequencies), and (iii) the critical frequency. However, we did not find any significant relationship (*p* > 0.05). Thus, the mere number of practice hours could not explain the coordination performance in this study.

### Handedness

Professional musicians show a reduced asymmetry in hand skill (Jäncke et al., 1997; Fujii and Oda, 2006) and brain structure (Amunts et al., 1997). Playing musical instruments requires skilled bimanual coordination, and musical practice improves non-dominant hand skills while reducing hand skill asymmetry (Fujii et al., 2010). In this study, the handedness score of the dancers (78.1 ± 14.7) tended to be smaller than that of the non-dancers (92.9 ± 8.4; *p* = 0.0502). This indicates that the dancers use their left hand more frequently in their daily life than the non-dancers. We suggest that dance technique practice may alter hand preference because street dance techniques require skilled bimanual coordination. However, we cannot rule out the possibility that the tendency of the smaller handedness score in the dancers was because of chance. It would be interesting for future studies to test whether dancers show reduced hand skill asymmetry. There may be a common characteristic between dancers and musicians in terms of how the motor learning of the performing arts alters the human brain and behaviors.

### Effects of Musical Practice, Sex, and Age

Besides the effect of practicing street dance, musical practice is also thought to affect the performance of finger-to-beat coordination (for a review, Repp, 2005). A limitation of this study is that we could not strictly control the effect of musical practice (e.g., the types of musical instruments the participants had practiced and the amount of musical practice). Nevertheless, at least we counterbalanced the number of participants who had experience of instrumental practice: four dancers and four non-dancers had experience of practicing musical instruments. Thus, the group difference in the performance measures (i.e., the SD of the phase angle and the critical frequency) in the present study would ensure the effect of street dance practice. One of the interesting questions for future studies is to differentiate the effects of musical practice and street dance practice.

Another limitation of this study is that the participants of this study were men in their 20s and 30s. Whether the findings in this study can be generalized to other populations should be investigated in a future study.

## Conclusion

We investigated the finger-to-beat coordination of non-dancers, street dancers, and a world street-dance champion. We found that the dancers were able to maintain prescribed coordination patterns more stably at the high beat rates even when they only moved the distal part of their body. These results suggest that the skill of accomplished dancers not only lies in big knee movements but also in small finger movements. The phrase “God is in the details” may apply to street dance. These findings provide additional evidence that the sensorimotor learning of rhythmic dance is characterized by a stabilization of the coordination pattern, including the inhibition of an unintentional transition to other coordination patterns. We also found that the world champion was able to match the timing of the movement peak velocity to the beat across the different beat rates. This would help understand the characteristics of highly organized street dance techniques.

## Author Contributions

AM, KK, and KN conceived and designed the experiments. AM performed the experiments. AM, SF, and MO analyzed the data. All authors contributed to and have approved the final manuscript.

## Conflict of Interest Statement

The authors declare that the research was conducted in the absence of any commercial or financial relationships that could be construed as a potential conflict of interest.

## Footnotes

1 In this paper, “coordination" refers to a spatiotemporal organization (pattern formation) between the movements of the limbs, or between movement and environmental information (auditory or visual stimuli). On the other hand, “synchronization" refers only to the phase locking relationship between two components (e.g., movement and beat). For example, Kelso (1995) used the two terms as follows: “In our experiment the task for the subject was to *synchronize* peak flexion of the index finger with a metronome in two modes of *coordination*: on the beat and off the beat." (p. 102).

2 Street dance was originally developed by African-Americans (Shichirui, 1999). The main characteristics of street dance is to coordinate whole-body movements with musical beats whose rate is approximately 100 beat per minute or more.
